# Temporal Differences in MicroRNA Expression Patterns in Astrocytes and Neurons after Ischemic Injury

**DOI:** 10.1371/journal.pone.0014724

**Published:** 2011-02-23

**Authors:** Mateo Ziu, Lauren Fletcher, Shushan Rana, David F. Jimenez, Murat Digicaylioglu

**Affiliations:** Department of Neurosurgery, University of Texas Health Science Center at San Antonio, San Antonio, Texas, United States of America; City of Hope Comprehensive Cancer Center, United States of America

## Abstract

MicroRNAs (miRNAs) are small, non-protein-coding RNA molecules that modulate gene translation. Their expression is altered in many central nervous system (CNS) injuries suggesting a role in the cellular response to stress. Current studies in brain tissue have not yet described the cell-specific temporal miRNA expression patterns following ischemic injury. In this study, we analyzed the expression alterations of a set of miRNAs in neurons and astrocytes subjected to 60 minutes of ischemia and collected at different time-points following this injury. To mimic ischemic conditions and reperfusion *in vitro*, cortical primary neuronal and astrocytic cultures prepared from fetal rats were first placed in oxygen and glucose deprived (OGD) medium for 60 minutes, followed by their transfer into normoxic pre-conditioned medium. Total RNA was extracted at different time-points after the termination of the ischemic insult and the expression levels of miRNAs were measured. In neurons exposed to OGD, expression of miR-29b was upregulated 2-fold within 6 h and up to 4-fold at 24 h post-OGD, whereas induction of miR-21 was upregulated 2-fold after 24 h when compared to expression in neurons under normoxic conditions. In contrast, in astrocytes, miR-29b and miR-21 were upregulated only after 12 h. MiR-30b, 107, and 137 showed expression alteration in astrocytes, but not in neurons. Furthermore, we show that expression of miR-29b was significantly decreased in neurons exposed to Insulin-Like Growth Factor I (IGF-I), a well documented neuroprotectant in ischemic models. Our study indicates that miRNAs expression is altered in neurons and astrocytes after ischemic injury. Furthermore, we found that following OGD, specific miRNAs have unique cell-specific temporal expression patterns in CNS. Therefore the specific role of each miRNA in different intracellular processes in ischemic brain and the relevance of their temporal and spatial expression patterns warrant further investigation that may lead to novel strategies for therapeutic interventions.

## Introduction

The discovery of microRNAs (miRNAs), a novel class of small non-protein-coding molecules, has introduced a whole new level and mechanism of gene regulation[1]–[5]. Long RNA precursors of miRNAs are transcribed from the cellular genome and undergo sequential processing by several RNase complexes to produce small (18 – 25 nucleotides) mature miRNAs[6]. MiRNAs regulate gene expression at the post-transcriptional level by altering translation of target messenger RNAs (mRNAs) into proteins. The mechanisms by which miRNAs function is by inhibition of the protein translation or by promotion of mRNA decay[7]. By targeting the mRNA of protein-coding genes, miRNAs play a critical role in development, control of cell growth, proliferation, metabolism and in a variety of other biological processes[6]. MiRNAs have been implicated as well in the etiology of a variety of pathological processes such as cancer, ischemia, developmental arrest, inflammatory diseases and cellular response to stressful conditions [7]–[37].

Ischemia is an essential feature of Central Nervous System (CNS) injury in stroke, cancer, traumatic brain injury (TBI) and spinal cord injury (SCI). Neurons and astrocytes, as other cells respond to injury by altering their gene and protein expression patterns[38], [39]. Since the first discovery that miRNAs are present in the CNS[4], several studies have suggested miRNA's involvement in CNS physiological and pathological processes[1], [5], [22]–[37], [40]–[42]. Expression profiling of miRNA at the tissue level has been performed in stroke, TBI and SCI. In these studies it has been reported that miRNAs demonstrate significant expression level changes, suggesting their implication in the disease process[32], [35], [37]. Presently, little is known about the alteration of miRNAs levels in neuronal and astrocytic response to ischemic conditions[32]. A better understanding of the molecular mechanisms responsible for the behavior of the different CNS cells during ischemic injury may lead to novel strategies for cell-specific therapeutic interventions.

The CNS has different cell types with different roles that react differently to changes in the surrounding environment. In this context, miRNA profiling from whole brain tissue cannot relate expression changes to a certain cell type and distinguish cell-specific patterns in neurons and astrocytes. This shortcoming of miRNA profiling experiments from the tissue level can impair our ability to draw accurate conclusions on miRNA cell-specific role[43]. Thus, to understand the role of miRNAs in the cerebral injury, there must first be an understanding of how different miRNAs behave in different cell types involved in a particular pathological process. We hypothesized that miRNA expression patterns differ in astrocytic and neuronal cells in response to ischemic conditions.

From the current literature, we identified a set of miRNAs that have been reported to alter their expression pattern during TBI and stroke at the tissue level. These miRNAs appear to play an important role during hypoxic stress in other non-cerebral cell types as well[7], [44]. In this study, we compared temporal changes in expression levels of this set of miRNAs that were induced by oxygen-glucose deprivation (OGD) in astrocytes and neurons. We report here that in neurons and astrocytes, miRNAs alter their expression levels in response to OGD *in vitro* and that these alterations are cell-specific. Furthermore, we show that a known neuroprotectant, Insulin-Like Growth Factor I (IGF-I), can reduce the expression of miR-29b, which is highly upregulated in ischemic neurons.

## Materials and Methods

### Cell cultures

All the animal work was carried out in the animal facility at the University of Texas Health Science Center in San Antonio (UTHSCSA) after being approved by the Animal Care and Use Committee at the UTHSCSA (Protocol Number 07071) and adhered to the National Institute of Health principles of laboratory animal care (NIH publication No. 80–23).

Mammalian cortical neurons were grown in dissociated cultures as previously described[45]–[47]. Briefly, pregnant Sprague-Dawley rats, 17 days post conception, were sedated and embryos were extracted with sterile technique. The brain cortices of the embryos were removed, and after trypsinization, cells were plated onto poly-L-lysine coated 35 mm dishes and maintained in serum-free Neurobasal Medium supplemented with B27 at 37°C, 5% CO2, in a humidified environment. Astrocyte cultures were isolated from 17 day-old rat embryos as well, and maintained in DMEM containing 10% bovine calf serum at 37°C, 5% CO2 in a humidified environment and allowed to grow to confluence. Astrocytes were then trypsinized and plated onto 60 mm dishes at a density of 1.5×10^4^ cells/cm^2^ and used when they were confluent. The purity level of each culture was tested using immunofluorescent techniques. Cells grown on cover slips were labeled with a neuronal marker (anti-MAP2, Sigma) and an astrocyte marker (anti-GFAP, Sigma). Neuronal cultures contained less than 3% of GFAP-positive cells. Astrocytic cultures did not contain any MAP2-positive cells. Four different litters were used for each set of experiments to produce an n = 4.

### Oxygen–glucose deprivation

Cells in culture were placed in oxygen-glucose deprivation (OGD) conditions as previously described[48]. Briefly, the culture medium was replaced by a glucose-free Earle's balanced salt solution (EBSS) with the following composition: KCl 5 mM, NaHCO3 26 mM, NaCl 117 mM, NaH2PO4 1 mM, which was previously saturated with 1% O2, 5% CO2, 94% N2 gas at 37°C overnight. The cultures were then placed in an airtight incubation chamber (CBS Scientific) equipped with inlet and outlet valves, and flushed for 4 minutes with a continuous influx of 1% O_2_, 5% CO_2_, 94% N_2_ gas at a flow rate of 20 L/minute. The chamber was then sealed to maintain the gas composition and placed into an incubator at 37°C for 60 minutes. Afterwards the cultures were removed from the airtight hypoxic chamber and the glucose-free EBSS medium was replaced with the pre-OGD conditioned medium. At 0, 2, 4, 6, 8, 12 and 24 hours after termination of OGD conditions the cells were collected and RNA extracted. Control cell cultures were not exposed to OGD. The experiments were performed in quadruplicate.

### MicroRNA collection

RNA was isolated from primary rat cultures that were highly purified (>98%) for either cerebral cortical neurons, or astrocytes that had undergone OGD or were maintained under normoxic conditions as described above. Cells were rinsed in PBS, lysed and scraped from the culture plates. Total RNA (including miRNAs) was isolated using the miRVana miRNA isolation kit according to the manufacturer's instructions (Ambion, Austin, TX). RNA quantification and quality assessment was performed by measuring the ultraviolet absorbance at 260 nm wave-length and the 260 nm/280 nm absorbance ratio in a DU530 spectrophotometer (Beckman Coulter)[33]. A ratio of 1.8–2.1 was considered satisfactory[33].

### TaqMan RT-PCR

For the quantitative analysis, two-step TaqMan real-time RT-PCR was performed using TaqMan MicroRNA Reverse Transcription kit (Applied Biosystems) on a 7500HT real-time PCR instrument (Applied Biosystems). Total RNA was converted into cDNA by reverse transcriptase (RT) reaction that was performed by sequential incubation at 16°C for 30 min, 42°C for 30 min, 85°C for 5 min and 4°C. The same amount of RNA for each sample was added to the RT reaction mix for each miRNA of interest. The input amount consisted of 10 ng of total RNA for each 15 µL of RT reaction as recommended by the manufacturer (Applied Biosystems, Protocol 4364031 Rev. E) and as suggested in the literature[28], [37]. PCR reaction mixture (20 µL) contained 1.33 µL of RT product, 10 µL of TaqMan 2X Universal PCR Master Mix and 1 µL of the appropriate TaqMan MicroRNA Assay (20X) containing primers and probe for the miRNA of interest (Applied Biosystems). The mixture was initially incubated at 95°C for 10 min, followed by 40 cycles of 95°C for 15 seconds and 60°C for 60 seconds. Primers for mature miR-21, miR-29b, miR-30b, miR-107, miR-137 and miR-210 (Applied Biosystems) were designed using the known mouse sequences from miRNA database[49]–[52] at http://www.mirbase.org (Table 1). Analysis was performed in duplicate with no-template control used as negative control.

**Table 1:** The Sequences of the Primers for miRNAs used.

miRNA name	Identification	Accession Number	Primers Sequence
miR-21	mmu-miR-21	MIMAT0000530	TAGCTTATCAGACTGATGTTGA
miR-210	mmu-miR-210	MIMAT0000658	CTGTGCGTGTGACAGCGGCTGA
miR-137	mmu-miR-137	MIMAT0000149	TTATTGCTTAAGAATACGCGTAG
miR-29b	mmu-miR-29b	MIMAT0000127	TAGCACCATTTGAAATCAGTGTT
miR-30b	mmu-miR-30b	MIMAT0000130	TGTAAACATCCTACACTCAGCT
miR-107	mmu-miR-107	MIMAT0000647	AGCAGCATTGTACAGGGCTATCA

Sequences were reviewed on miRNA database[49]–[52], [83] at http://www.mirbase.org.

SnoRNA-135, 18S rRNA, snoRNA-202, RN-U6 and human β-actin RNA were tested as internal controls on neurons and astrocytes. The expression levels of these small RNAs were altered in excess of 1.5 fold in at least one time-point (data not shown) in both neurons and astrocytes. It is a well-known phenomenon that experimental conditions may alter the expression of genes that are candidates for internal controls[34], [53], [54]. Hence, to analyze the data, we used the method described for these conditions by Livak and Schmittgen[55] where, after determining the concentration of RNA by UV absorbance method in a spectrophotometer[55], the same amount of RNA was converted into cDNA and used for each RT-PCR experiment. The relative expression of every specific miRNA for each time-point was compared to that of normoxic specimen and presented as 2^−Δ_C’T_^, (where ΔC’_T_ = C_T, Time x_ – C_T, Control_ and C_T_ is the cycle threshold)[55], [56].

### Insulin-Like Growth Factor I (IGF-I)

Recombinant human IGF-I (R&D Systems) was reconstituted at 100 µg/mL in sterile PBS as suggested by the manufacturer. Normoxic neuronal cultures (35 mm dishes) were treated with 100 ng/mL of IGF-I and changes in miRNA levels were measured using RT-PCR. We screened the same miRNAs as described above (miR-21, miR-29b, miR-30b, miR-107, miR-137, miR-210). Total RNA was isolated from neurons using the miRVana miRNA isolation kit. Two-step TaqMan real-time RT-PCR was performed using TaqMan MicroRNA Assays on a 7500 real-time PCR instrument as described above. All RNA samples were normalized to an internal control, U6 small nuclear RNA. Analysis was performed in duplicate, with no-template controls. Relative expression was calculated using the comparative cycle threshold method (2^−ΔΔ_Ct_^)[56]. All data is expressed as the mean ± the SE (n = 3).

### Statistical Analysis

Quantitative RT-PCR data were compared by one-way analysis of variance (ANOVA) followed by Dunnett's multiple comparison tests to determine overall significance of fold-changes in miRNA expression levels compared to control. Mean expression levels for each miRNA were compared for each time point between neurons and astrocytes using Student's t test. For correlation, R-squared values were calculated using the log of the fold changes in miRNA levels. P values of <0.05 were considered to be significantly different.

## Results

We examined temporal changes in expression levels of a specific set of miRNAs in neurons and astrocytes at 0 h, 2 h, 4 h, 6 h, 8 h, 12 h and 24 h after the OGD as compared to cells growing in normal conditions (control). We considered a trend of expression change when expression level was altered more than 1.5-fold, and strong expression change if altered >2-fold[8]. We chose these specific miRNAs because they have been shown to alter their expression levels in several CNS pathologies[31], [32], [35], [37], [38]. Furthermore these miRNAs have been reported to be involved in cell response to hypoxia and are considered to be part of a set identified as hypoxia-regulated microRNAs (HRMs)[7], [8], [44].

**Figure 1:**
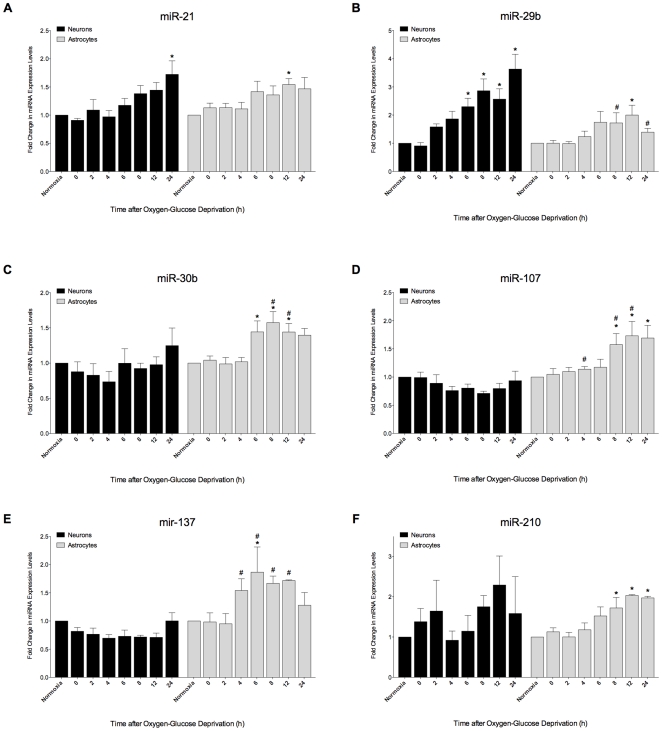
Effects of oxygen-glucose deprivation on miRNA expression in neurons and astrocytes. RNA was isolated from purified cortical neurons or astrocytes that had undergone OGD (1 h) or were maintained in normoxic conditions. RNA concentrations were determined by UV absorbance, and 10 ng for each sample was converted into cDNA and expression levels were measured using RT-PCR[55]. The fold-change in expression of each miRNA compared to that of the normoxic specimen was calculated using 2^−Δ_C’T_^ formula. Data is expressed as the mean ± the SE (n = 4). The results were analyzed by one-way ANOVA followed by multiple comparisons test (* p<0.05, normoxia versus OGD within each cell type). Differences in the mean expression level for each miRNA between the two different cell types was compared for each time point post-OGD by Student's t test (# p<0.05, astrocyte versus neuron).

MiR-21 was upregulated 1.5-fold at 12 h and 1.94-fold (p<0.05) at 24 h post-OGD as compared to expression in control neurons. MiR-29b showed the highest expression alteration in neurons exposed to OGD. It was upregulated by 1.6-fold at 2 h post-OGD (p<0.05), and 4-fold (p<0.05) at 24 h suggesting a strong involvement of this miRNA in the neuronal response to ischemia. MiR-30b, miR-107 and miR-137 did not alter their expression levels in neurons. MiR-210 was more than 2-fold upregulated at 12 h and 24 h (Figure 1).

A different pattern of expression for the miRNAs tested was noticed in astrocytes undergoing OGD (Figure 1). MiR-21 showed a trend of upregulation (>1.6-fold, p<0.05) only at 12 h post-OGD. MiR-29b expression was upregulated in astrocytes as well, but to a lesser magnitude than in neurons. MiR-29b showed a trend of upregulation at 6 h post-OGD with >1.7-fold increase, and >2-fold at 12 h post-OGD (p<0.05). MicroRNAs 30b, 107 and 137 in astrocytes expressed a change in their levels post-OGD conditions. MiR-30b showed a trend of expression change at 6, 8 and 12 h (p<0.05). MiR-107 was upregulated 1.56-fold at 8 h and 1.7-fold at 12 and 24 h (p<0.05). MiR-137 showed a trend of upregulation after 4 h post-OGD and continued to be upregulated as well at 6, 8 and 12 h. MiR-210 was upregulated after 6 h by >1.6-fold and significantly upregulated at 8, 12 and 24 h (p<0.05) suggesting a strong involvement of this miRNA in astrocytic response to ischemia (Figure 1).

**Figure 2:**
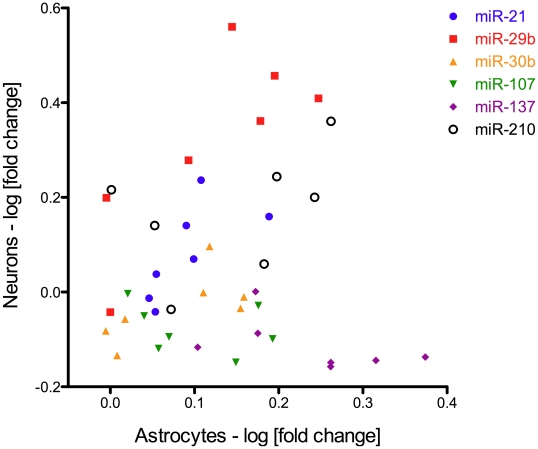
MicroRNAs express different alteration patterns in neuron and astrocytes undergoing OGD conditions. Each data point represents the average *log* of fold changes of a particular miRNA at different time-points in neurons and astrocytes from 4 independent experiments. Note that there is a very poor correlation (R^2^ correlation coefficient  = 0.098) between the miRNAs expressions in neurons and astrocytes at different time-points post-OGD.

In our study, we found that there is a poor correlation (R^2^ correlation coefficient  = 0.098) between expression levels of each specific miRNA in astrocytes and neurons in response to ischemic conditions at different time-points (Figure 2). While in neurons undergoing OGD conditions, miR-30b, 107 and 137 did not show any expression alterations, these same miRNAs showed a significant expression upregulation in astrocytes when compared to cells in normoxic conditions. MiR-29b is upregulated in both neurons and astrocytes post-OGD, but at different time-points and with remarkable difference in magnitude. MiR-21 is significantly upregulated at 12 h and 24 h in astrocytes however in neurons upregulation occurred only after 24 h. MiR-210 is significantly upregulated in astrocytes at 8 h, 12 h and 24 h. In neurons, miR-210 showed a significant variability between each set of experiments. This might suggest that miR-210 is tightly controlled in ischemic conditions in neurons and small changes in experimental conditions may affect its expression significantly. Unfortunately, no definitive conclusions could be drawn for miR-210 involvement in neurons exposed to OGD.

A direct comparison of expression levels of all the miRNAs used for our study between neurons and astrocytes undergoing OGD conditions, showed a significant difference at different time-points only for miR-29b, miR-30b, miR-107 and miR-137 (Figure 1).

**Figure 3:**
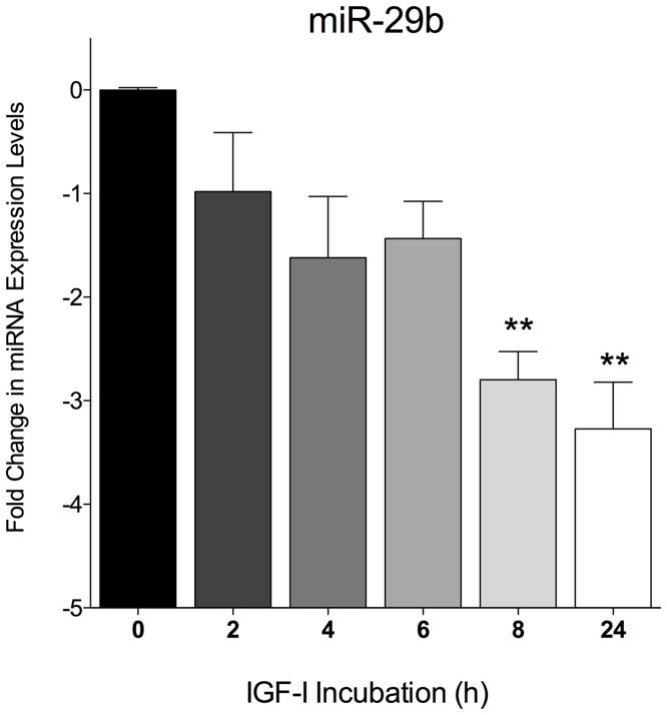
Insulin-Like Growth Factor I (IGF-I) temporally decreases microRNA-29b. Total RNA was isolated at various time points from neuronal cultures treated with IGF-I (100 ng/mL). All RNA samples were normalized to an internal control, U6 small nuclear RNA. Analysis was performed in duplicate, with no-template controls. Relative expression was calculated using the comparative cycle threshold method (2^−ΔΔ_Ct_^). Data is expressed as the mean ± the SE (n = 3). Results were analyzed by one-way ANOVA followed by multiple comparisons test (** p<0.01, control versus IGF-I treatment).

IGF-I has been shown to be neuroprotective in a number of ischemic models[47], [57]–[59]. We wanted to determine if a neuroprotectant, such as IGF-I, could effect the expression of miRNAs that are known to be altered under ischemic conditions. Therefore, neuronal cultures were exposed to IGF-I (100 ng/mL) for 24 h and changes in miRNA levels were measured at various time points using RT-PCR. We screened the same miRNAs as described above (miR-21, miR-29b, miR-30b, miR-107, miR-137, miR-210) and found that IGF-I significantly decreases the expression of mir-29b in neurons. Compared to control (0 h), miR-29b was decreased -2.8-fold at 8 h and was further decreases by -3.3-fold at 24 h (Figure 3). IGF-I did not have an effect on the expression levels of mir-21, mir-30b, mir-107, mir-137, or mir-210 in neurons.

## Discussion

It is believed that multiple post-transcriptional mechanisms play an important role in the regulation of gene expression[60]. It is becoming widely accepted that miRNAs are an important part of these regulatory pathways[1], [9], [25], [30], [32], [33], [37], [60]–[63].

Ischemia is a hallmark of cellular stress and is considered one of the major culprits of CNS injury in many pathological processes. It has been suggested that miRNAs play an important role in cellular response to hypoxic stress[8], [12], [64], [65]. Kulshreshtha and colleagues[12] have identified a set of hypoxia-regulated microRNAs (HRM) in tumor cells, providing an additional link between tumor-specific stress factors and gene expression control. Furthermore, miRNAs have been shown to change their expression levels in myocardiocytes during ischemic stress[66]. It has been recently reported that expression of miRNAs is altered in the injured CNS tissue following stroke, TBI and SCI[32]–[35], [37]. To our knowledge the relation between cell-specific temporal changes in expression levels of miRNAs and hypoxia *in vitro* has not been reported in neurons and astrocytes.

Our study demonstrates that expression levels of miRNAs change when neurons and astrocytes are subjected to OGD, suggesting miRNA involvement in the cellular response to ischemia in these cells. Furthermore we show that miRNA temporal expression alterations in response to OGD are different in astrocytes and neurons. We found that immediately after 60 minutes of ischemia (time 0 in our experiments), none of the miRNAs studied demonstrated a change in their expression levels in neither cell type (Figure 1). Three possible explanations could be considered: First, after 60 min of OGD, neurons and astrocytes may utilize the remaining resources for basic cell-survival needs and not in activation and transcription of miRNAs, and gene translation into proteins. Second, it could be that synthesis of miRNAs is a later event in the hypoxic cascade of these cells response to ischemia. Lastly, other miRNAs may be involved in the early response of neurons and astrocytes to ischemia, and the miRNAs studied here are involved at later time – points. At 2 h, we saw a trend of upregulation of miR-29b in neurons that steadily increased up to 4-fold at 24 h. In contrast, in astrocytes, miR-29b showed a trend of upregulation later, after 6 h and a significant level increase by 2-fold only after 12 h. MiR-30b, miR-107 and miR-137 were uniquely upregulated only in astrocytes at different time-points, starting 6 h after OGD event. MiR-210 showed a trend of upregulation after 6 h and a significant upregulation after 8 h in astrocytes (Figure 1). MiR-21 showed a similar expression alteration in neurons and astrocytes. MiR-29b, miR-30b, miR-107 and miR-137 showed a significant difference in their expression levels between neurons and astrocytes undergoing OGD conditions (Figure 1). Furthermore, from our experiments, we discovered that the 6 h mark appears to be important for different miRNAs to alter their expression levels in neurons and astrocytes after ischemic injury.

Wang and colleagues[67] have shown that there is a difference in the miRNA repertoire between superficial white matter and overlying gray matter in humans. In our study, we demonstrate that OGD induces miRNA expression in a cell-specific pattern at different time-points in astrocytes and neurons. We speculate that this could be secondary to the difference in the baseline cell specific repertoire of miRNAs, or considering their role and function in the global physiology of the brain, astrocytes and neurons activate different biochemical pathways after ischemia and hence different miRNAs are expressed in each cell type.

MiRNAs modulate gene expression at the posttranscriptional level and can modulate many different cellular and pathological processes by fine tuning protein expression patterns[35]. It is thought that ischemia induces genes that cause apoptosis[68]. Demonstrating that astrocytes and neurons express cell specific changes in miRNA levels after ischemic injury, is a significant step toward understanding of their involvement in the underlying molecular mechanisms of cerebral injury. Park and colleagues[69] have shown that family members of miR-29 upregulate the expression of p53 through regulation of transcription of cdc42 and p85α in tumor cells and as such induce apoptosis. Garzon and colleagues[70] reported that over-expression of synthetic miR-29b in acute myeloid leukemia (AML) cell lines and primary AML blasts induced apoptosis. These studies strongly support the idea that by the blockage or enhancement of miR-29b expression we could influence apoptosis of cells of interest[69], [70]. Control of neuronal apoptosis after ischemia is important when considering that the degree of neurologic disability after CNS hypoxic injury depends on the extent of neuronal loss. In this study we were able to show a novel effect of IGF-I, that of suppression of miR-29b expression in neurons. We speculate that the downregulation of miR-29b by IGF-I could possibly play a role in the anti-apoptotic signaling afforded by activation of the IGF-I receptor, but further studies would be needed in order to substantiate this hypothesis.

Another recent study found that miR-29 family could regulate DNA methylation via DNA methyltransferase 3A and 3B transcriptional regulation[71]. Abnormal DNA methylation can cause cell death and has been documented to be altered in Alzheimer disease. In addition miR-29a/b was shown to be involved in BACE-1 expression in patients with Alzheimer's disease[31]. Our findings that miR-29b is upregulated in astrocytes and neurons after ischemia *in vitro* opens the path for further investigations to the role of miR-29 in apoptosis in other forms of dementia that have been related to lacunar brain ischemia. Hypoxia has been shown to induce the upregulation of miR-210 in cancer and non-cancer cells[7], [12]. Zhang and colleagues[72] have recently described that miR-210 influences the hypoxic response in tumor cells through targeting a key transcriptional repressor of the MYC-MAX network. In our study, miR-210 is significantly upregulated in astrocytes after 8 h.

While our efforts in neurons are focused on finding those miRNAs involved in apoptosis after ischemic insult; in astrocytes it is important to study those miRNAs that may control their function in protecting the CNS environment after ischemia. It has been postulated that miRNAs are involved in inflammation and cell response to free radicals after hypoxia[73]. MiR-21 alteration has been shown to be important in protecting myocardiocytes against H_2_O_2_ radicals after ischemia[74]. MiR-21 is upregulated in astrocytes 12 h after ischemia, the time when multiple free radicals are present in ischemic brain. The role of miR-21 in the cellular response to the free radicals in CNS warrants further investigation. MiR-107, miR-30 and miR-137 were upregulated only in astrocytes. While it is premature to anticipate any specific role for these miRNAs in astrocytic response to ischemic conditions *in vitro,* few speculations can be made based on their suggested role in other cellular systems of recent investigation.

Progranulin/granulin (GRN) is a protein that modulates inflammation and gliosis. A recent study by Wang and colleagues[75], indicated that miR-107 contributes to *GRN* expression regulation in human H4 neuroglioma cells and a TBI rat model. It can be speculated that changes in expression levels of miR-107 in astrocytes after OGD, may modulate inflammation and gliotic processes in the brain after hypoxic injury through alteration of GRN expression. It has been reported that miR-137 expression is decreased in astrocytoma cell lines relative to normal brain tissues[76]. In this study, miR-137 was found to be a direct inhibitor of CDK6, suggesting a role of this miRNA in astrocytoma cell proliferation. In light of these results, our findings that miR-137 level is increased only in astrocytes exposed to OGD-conditions, suggest that miR-137 may induce astrocytic proliferation as observed after ischemic stroke[77], [78]. To our knowledge, the role of miR-30 has not been studied in astrocytes. Recently it was reported that members of miR-30 family are involved in myocardial extracellular matrix remodeling targeting connective tissue growth factors that are induced by TGF-β and endothelin[79]. It is known that TGF-β and endothelin-1 regulate astrocyte proliferation and reactive gliosis as well [80], [81]. It is tempting to speculate a role of miR-30 in controlling apoptosis in astrocytes under hypoxic stress conditions and in induction of astrocyte proliferation and reactive gliosis through similar pathways as in cardiomyocytes.

Jeyaseelan and colleagues[32] reported that expression of several miRNA were altered at 24 h and 48 h after reperfusion in brains of adult rats subjected to transient focal ischemia by middle cerebral artery occlusion (MCAo). They found that miR-21 and miR-210 expression are upregulated at 24 h after reperfusion that is in line with our findings in neurons and astrocytes exposed to OGD conditions. Similarly, in accord with our findings, Rendell and colleagues[35] reported upregulation of miR-21 and miR-30b occurring 24 h after TBI in brain tissue in a rat model. In line with our findings in cell cultures, Liu and colleagues[34] also found an increase in expression levels of miR-107 (at 24 h) in brain tissue of an intraparenchymal hemorrhage adult rat model, while Rendell[35] reported a downregulation of miR-107 (at 3, 24, 72 h) in the TBI adult rat model. Differently from Jeyasselan[32] study in transient ischemia model and our study in cell cultures subjected to OGD conditions, Liu[34] found a 1.6 fold downregulation of miR-210 in the hippocampus of a transient ischemia of MCAo rat model. In his study, Dharap[28] reported miR-29b (at 3, 6 and 24 h) and miR-137 (at 24 h) to be downregulated in a MCAo adult rat model, while Lei[33] reported the same findings in a TBI adult rat model at 6 h post injury. When comparing our finding of miRNA expression changes in neuron and astrocyte cell cultures subjected to OGD conditions with changes reported in brain tissue subjected to ischemic conditions several considerations need to be made. Most of these studies were made in adult rat brains. Furthermore some of them evaluated miRNA expression changes in brain cortex[33], some in hippocampus[35] and others in the entire brain tissue[32], [34]. In contrast, our experiments were performed on cultures of neurons and astrocytes prepared from cortex of rat embryos. Studies have shown that while some miRNAs are expressed during neuronal differentiation, others are expressed only in mature neurons[41], [82] assisting in explaining some of the differences found between our results in embryonic neurons and astrocytes cultures and those reported by others in adult brain tissue. Another consideration to be made is on the differences in the methodologies used to isolate and detect the different miRNAs. Several reports and reviews have pointed out the differences that exist between different microarray platforms that are in use for miRNA profiling, RT-PCR techniques, and different miRNA isolation kits in today's market from different vendors that can interfere with our ability in comparing our results with other reports[67]. Lastly, it should not be forgotten that the 3D configuration of the brain tissue allows for a substantial difference in the communication between different cells in the space and their influence on each other, when compared to monolayer cell cultures' single type cell interaction.

We have shown here that the induction of changes in miRNA expression levels is a normal response to ischemia in neurons and astrocytes and not necessarily only in cancerous and myocardial cells[7], [8], [25], [74]. We found significant differences in the onset and magnitude of induction for individual miRNAs in neurons and astrocytes in response to ischemia. Furthermore, we demonstrate here that astrocytes and neurons express different miRNAs at different time-points due to their difference not only in their miRNA repertoire, but also in their cell specific function within the CNS. Therefore we suggest that different miRNAs are involved at different time-points in order for these cells to activate different biochemical pathways after ischemia to better accomplish their functions.

In conclusion, our study adds further evidence that hypoxia induces time-dependent alteration of miRNAs expression levels suggesting their involvement in the cellular response to ischemic injury. Furthermore, we provide evidence that in CNS, different cells activate different miRNAs and at different time-points in response to ischemic conditions. Other studies that would utilize the microarray technology could discover other miRNAs that show expression alteration after OGD in neurons and astrocytes. Elucidation of the role of miRNAs in ischemia and identification of their specific targets in astrocytes and neurons warrants further investigation that may lead to new therapeutical strategies in CNS injury.
